# Toward personalised diffusion MRI in psychiatry: improved delineation of fibre bundles with the highest-ever angular resolution in vivo tractography

**DOI:** 10.1038/s41398-018-0140-8

**Published:** 2018-04-25

**Authors:** Fraser Callaghan, Jerome J. Maller, Thomas Welton, Matthew J. Middione, Ajit Shankaranarayanan, Stuart M. Grieve

**Affiliations:** 1 Sydney Translational Imaging Laboratory, Heart Research Institute, Charles Perkins Centre and Sydney Medical School, University of Sydney, Sydney, NSW Australia; 2 General Electric Healthcare, Richmond, VIC Australia; 3 Applied Sciences Laboratory West, GE Healthcare, Menlo Park, CA USA; 4 Macquarie Medical Imaging, Macquarie University Hospital, Sydney, NSW Australia

## Abstract

Diffusion MRI (dMRI) tractography is a uniquely powerful tool capable of demonstrating structural brain network abnormalities across a range of psychiatric disorders; however, it is not currently clinically useful. This is because limitations on sensitivity effectively restrict its application to scientific studies of cohorts, rather than individual patients. Recent improvements in dMRI hardware, acquisition, processing and analysis techniques may, however, overcome these measurement limitations. We therefore acquired the highest-ever angular resolution in vivo tractographic data set, and used these data to ask the question: 'is cutting-edge, optimised dMRI now sensitive enough to measure brain network abnormalities at a level that may enable personalised psychiatry?' The fibre tracking performance of this 'gold standard' data set of 1150 unique directions (11 shells) was compared to a conventional 64-direction protocol (single shell) and a clinically practical, highly optimised and accelerated 9-min protocol of 140 directions (3 shells). Three major tracts of relevance to psychiatry were evaluated: the cingulate bundle, the uncinate fasciculus and the corticospinal tract. We found up to a 34-fold improvement in tracking accuracy using the 1150-direction data set compared to the 64-direction data set, while 140-direction data offered a maximum 17-fold improvement. We also observed between 20 and 50% improvements in tracking efficiency for the 140-direction data set, a finding we then replicated in a normal cohort (*n* = 53). We found evidence that lower angular resolution data may introduce systematic anatomical biases. These data highlight the imminent potential of dMRI as a clinically meaningful technique at a personalised level, and should inform current practice in clinical studies.

## Introduction

Convergent evidence shows that diffusion MRI (dMRI) can detect abnormalities in the white matter tracts across a range of psychiatric disorders; however, there is still no clinically actionable imaging measure using dMRI tractography in psychiatry, with usage of this technique limited to research studies of cohorts of around 30–100 patients. Since recent technology has dramatically improved the quality and amount of information we can obtain from dMRI, there is reason to believe that there may now be sufficient signal to employ dMRI as a clinically relevant psychiatric tool, potentially enabling an imaging-driven personalised psychiatric approach. Here, we test this hypothesis using data from the highest-ever resolution dMRI data set.

Psychiatry is currently in the process of a redesign, moving from a paradigm of symptom description to a more mechanism-driven approach^1–3^. Advances in genetics, molecular biology and neuroimaging have enabled development of increasingly sophisticated models describing the major psychiatric disorders in terms of brain circuits, molecular dysfunction and genetic susceptibility. Despite these considerable advances, we are still unable to diagnose common psychiatric disorders with a single definitive test, and instead rely on complex composite criteria which draw on clinical, cognitive and other objective measurements. In contrast, some other areas of medicine have the luxury of early patient triage via highly sensitive and specific tests, greatly simplifying treatment algorithms and enabling development of early therapeutic options. An example of such a situation is angina, where cardiologists are able to objectively locate and quantify the pathological cause of a patient’s pain (occlusion of a coronary vessel) using a single test (coronary angiogram), before proceeding along an evidence-based treatment pathway informed by the severity and location of the lesion. The development of a similarly sensitive and specific single test that could map aberrant brain networks would greatly assist psychiatric care. Since the brain is fundamentally a network of interconnected regions, it therefore seems logical that accurate MRI 'connectomic' measurements of neural networks may provide the 'coronary angiogram equivalent' for psychiatry^4,5^.

The ability of dMRI to measure brain networks is based on the sensitivity of this technique to white matter tract microstructure. Water diffuses more freely along axonal fibres than across them and, through careful measurements of the diffusion rate in different directions, we are able to reconstruct detailed maps of anatomy^6^. Measurements of brain networks from dMRI have been used to generate fundamental insights across a range of conditions as varied as schizophrenia, depression, attention hyperactivity disorder and mild cognitive impairment^7^. A common limitation of current approaches, however, is that none are sufficiently specific and sensitive to guide clinical action at a personalised level, i.e., in individual patients. Our group recently published the first description of the precise brain circuits that characterise a diagnosis of depression^8^. This key advancement highlights the potential of dMRI as a clinical tool in psychiatric disease, but the data quality at the time necessitated a sample size of 100. In order to transition dMRI toward being a tool capable of informing a personalised medicine approach, much more precise and accurate measurements of brain structural abnormalities are required.

Early dMRI studies used a limited number of diffusion directions (e.g., 6 or 12), while more recent studies generally acquire a greater number. The majority of current studies use 32–64 directions. Numerous publications have evaluated the accuracy of fibre measurements using dMRI tractography. The majority of these have employed a low number of diffusion directions and used 'single-shell' acquisition schemes. The fibre tracking from such data is almost certainly dominated by the presence of spurious or underestimated neural pathways, necessitating large sample sizes to demonstrate group differences. These schemes are unable to resolve important differences between fibre tracks in terms of the precise shape, number and the degree to which they cross and 'kiss' other bundles. A higher number of unique directions (angular resolution) and shells will more confidently resolve inter-individual differences in white matter bundle density and anatomical course.

In this paper, we aimed to test the upper technological limit in overcoming these limitations by acquiring a state-of-the-art 'gold standard' data set at the highest-ever angular resolution dMRI (1150 unique diffusion directions). We then compared the performance of this data set in tracts relevant to psychiatry and compared these data to a configuration typical for current studies.

## Materials and methods

### Subjects

Fifty-three normal subjects drawn from the control group of the Chronic Diseases Connectome Project (CDCP) underwent an MRI assessment at Macquarie Medical Imaging, Sydney, NSW, Australia. One 42-year-old male subject had the additional 'gold standard' diffusion measurements. Subjects had no history of a psychiatric, neurological or cardiac disorder, and no contraindication to MRI scanning. The study had Institutional Ethics approval and all participants provided informed consent.

### Image acquisition

All imaging was performed using a 3 Tesla GE Discovery MR750w MRI scanner (GE Healthcare, Milwaukee, WI) with DV25.1 software and a 32-channel Nova head coil. dMRI data were acquired using a multiband, multi-shell spin-echo EPI pulse sequence. An initial dMRI data set was acquired using a highly optimised, clinically practical protocol, which was 140 diffusion directions comprising 25, 40 and 73 directions across three shells with *b *values of 700, 1000 and 2800 mm/s^2^, respectively (TR = 4323 ms, TE = 91.8 ms, flip angle = 90/180°, matrix = 128 × 128, field of view = 256 mm, voxel dimensions = 2 mm isotropic). A multiband factor of 3 was used, resulting in 66 acquired slices. To correct for eddy currents and distortions, a further diffusion-weighted sequence was also acquired (six directions) with a reversed phase-encoded (blipped) non-diffusion-weighted (*b* = 0) volume and a phase offset applied to each multiband component.

The 'gold standard' 1150-direction data set comprised nine consecutive diffusion-weighted sequences, which were then spliced together. Among these, the number of diffusion directions (ranging from 121 to 138), diffusion shells (ranging from 3 to 5) and *b* values (ranging from 500 to 2800 mm/s^2^) varied systematically across acquisitions to enable a final resolution of 1150 directions over 11 shells (Supplemental Table 1). The diffusion schemes were based on those proposed by Jones et al.^9^, in which distributions of data points are evenly distributed over a unit sphere in q-space. The same TE, TR, matrix, field of view and voxel dimensions were used for all dMRI scans. Overall, scanning time was 9 min for the 140-direction data set and 90 min for the full 1150-direction data set. The 64-direction data set was extracted from a single shell of the 140-direction data (*b* = 2800; see Supplemental Table 1).

Contiguous AC-PC aligned sagittal MPRAGE T1-weighted data with prospective motion correction (PROMO) were acquired with the following parameters: TR = 10.17 ms, TE = 4.06 ms, TI = 500 ms, flip angle = 8°, matrix = 284 × 284, field of view = 256 mm, voxel dimensions = 0.9 mm isotropic.

### Data pre-processing

Raw data were unaliased using an in-house MATLAB script (The Mathworks, Natick, USA). The first volumes (*b* = 0) from the first sequence and the blipped sequence were extracted and processed to remove geometric distortions^10^. The data sets were then merged into a single file to yield a 1150 diffusion direction data set. Eddy current correction was then performed on the merged data and each data set was inspected to ensure sufficient data quality^11^.

### Tractography

**Fig. 1:**
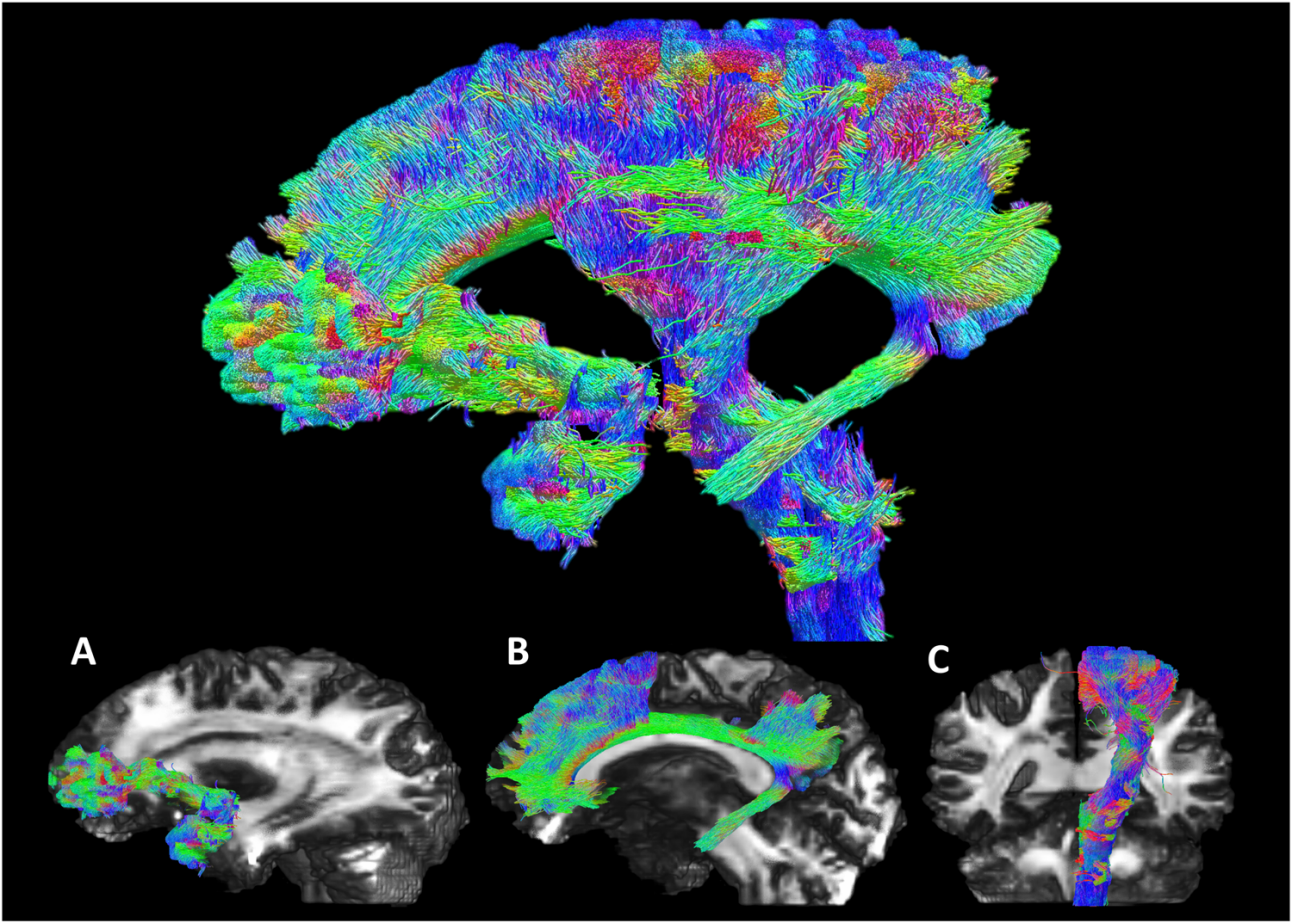
Appearance of the three selected master tracts presented together (top), and individually (bottom). Data are overlaid on the 1150-direction FOD amplitude image. (Bottom left) Uncinate fasciculus, (bottom middle) cingulate bundle, (bottom right) corticospinal tract. Streamlines are coloured by direction as per normal convention: superior–inferior in blue, anterior–posterior in green and left–right in red

**Fig. 2:**
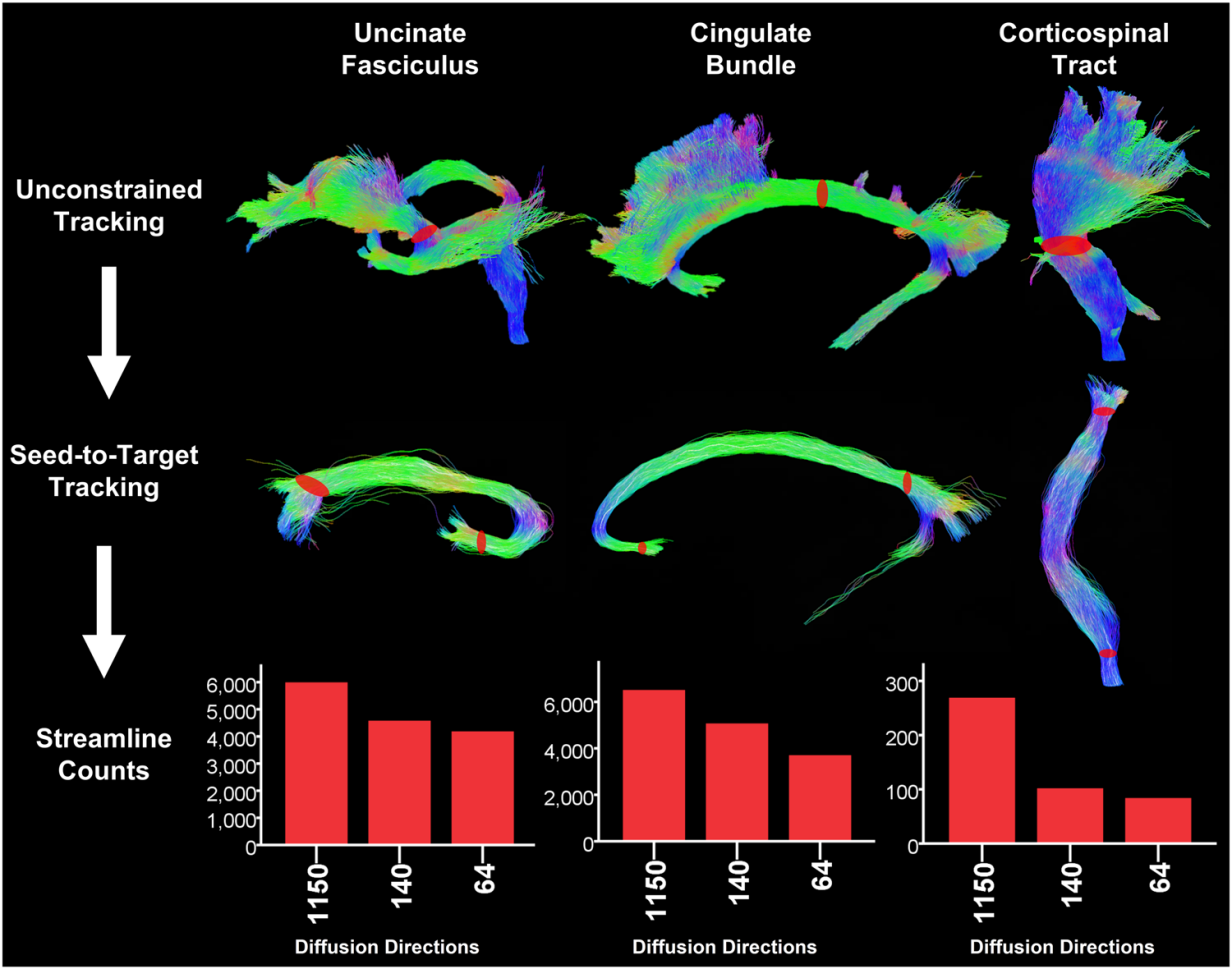
Improvement in tracking efficiency measured using source-to-target tracking. Seed volumes used for unconstrained tracking are overlaid on the resulting 'dominant bundle' tractograms from the 1150-direction data set (first row, centre of each tract). The source/target ROI definitions at the ends of each tract are overlaid on the corresponding tractograms from the 1150-direction data set (middle row). Bar charts display the number of successful streamlines to reach a target from 1 million source seeds (bottom)

To avoid the superposition of laterality effects, only left-sided tracts were analysed. We manually defined seed volumes at central locations of three major fibre bundles: uncinate fasciculus (UF), cingulate bundle (CB) and corticospinal tract (CST; Figs. 1 and 2). Seed-based tractography was performed in the 1150-direction data set, seeding 10 million streamlines bidirectionally using the MRtrix3 CSD iFOD2 technique for each of the seed volumes^12^. No exclusion, or target zones were used, allowing unconstrained propagation through the WM. Tracking was terminated when the angle between successive steps exceeded 45° or the streamline exited the brain volume. Tracks less than 20 mm in length were excluded. Track density images were created for each of the three bundles, to which we applied a threshold to retain only the 5% most densely tracked voxels, resulting in a gold standard 'dominant bundle'. The track density images were also used to define target and source regions of interest (ROIs; for use in the tracking efficiency test) at distal locations of each of the three bundles (Fig. 2 shows the analysis method).

The following outcomes were tested in the 64-, 140- and 1150-direction data sets:

- As a general measure of tracking performance (the tendency of the data to track, regardless of the accuracy), we measured the number of seed attempts required to successfully generate 1 million streamlines from each seed volume.
- To test the accuracy of tracking, we measured the number of streamlines that either remained inside ('dominant streamlines') or exited ('non-dominant streamlines') the dominant bundle. Non-dominant streamlines should consist of both anatomically correct and spurious fibres, and as tracking accuracy improves, one would expect an improvement in the dominant-to-non-dominant ratio approaching that of the gold standard ratio from the 1150-direction data set. We also measured streamline lengths for dominant and non-dominant streamlines.
- As a pure test of tracking efficiency, we measured the number of seed attempts required to generate 1000 streamlines between each target/source pair of ROIs in both directions. We also recorded the number of streamlines out of 1 million seeds that tracked from each source to the corresponding target.
- Finally, we performed a focussed analysis of tracking along the CB to test performance with increasing track complexity and branching. Four uniformly spaced target ROIs were placed along the cingulate between the posterior cingulate seed and the genu, and one in the frontal pole to measure branching.

To evaluate generalisability, we repeated the source–target and focussed CB analyses (outcomes c and d) in 64-direction and 140-direction data sets in 53 healthy individuals from the CDCP cohort. Streamline counts were compared using two-tailed *t*-tests.

## Results

### Visual improvement in delineation of tracts

Figure 1 shows tractograms of the single-subject 1150-direction data, highlighting the tracts isolated in the left hemisphere. The lower row shows the three major fibre bundles (UF, CB and CST) in isolation. The tracts conform closely to the expected anatomical appearance, with only very few spurious fibres visible.

**Fig. 3:**
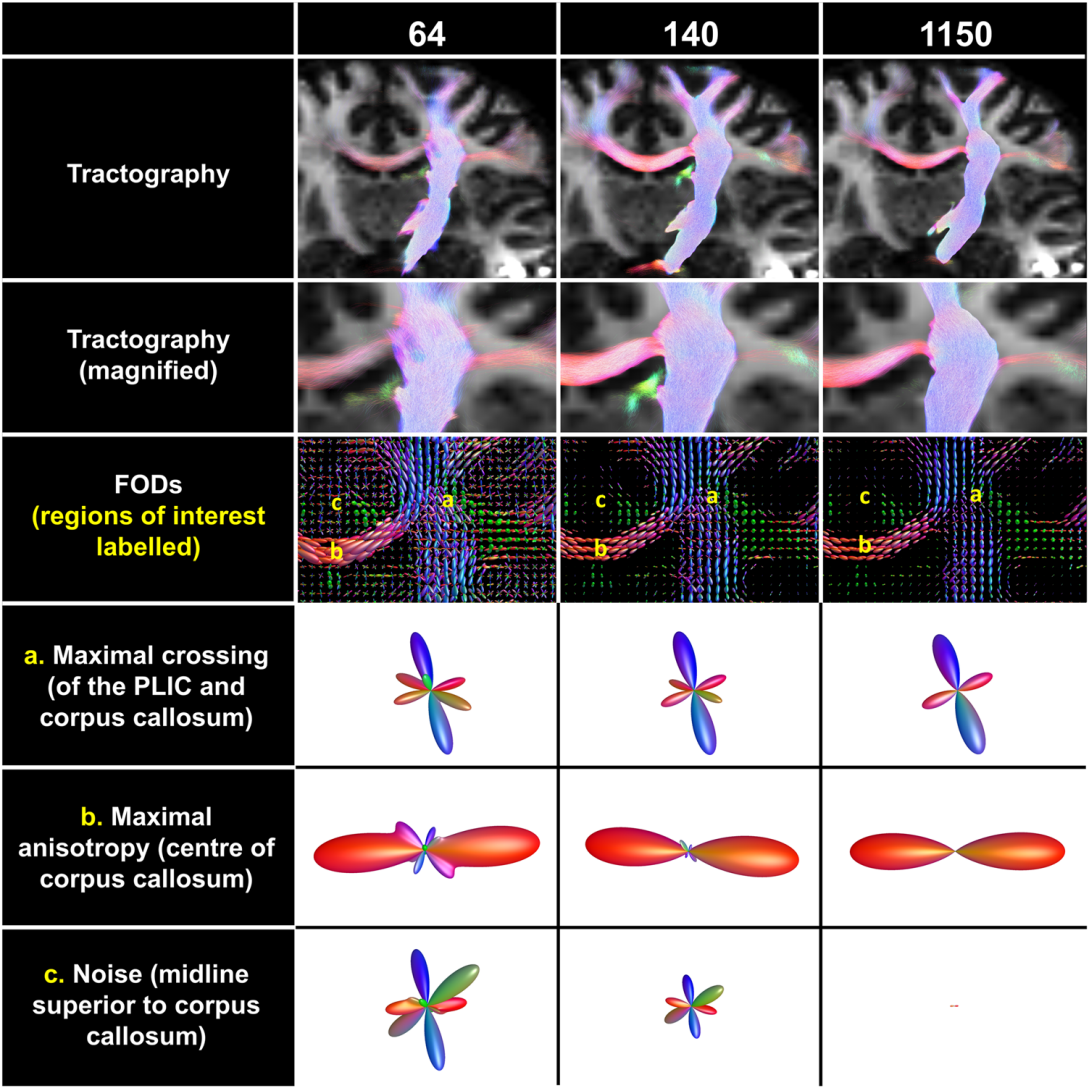
Comparison of improved tracking in the corticospinal tract and corpus callosum at high-angular resolution from 64 directions, 140 directions and 1150 directions. The top row shows a field of view in the coronal plane selected to display the course of the tract. The second row shows a magnified view at the level of the decussation of the contralateral fibres into the rostrum of the corpus callosum. The third row shows a view at the same level using a FOD representation of the data. The fourth row shows a single representative FOD from a region with maximal crossing fibres (**a**). The fifth row shows a single representative FOD from a region in the corpus callosum with maximal collinear fibres (anisotropy) (**b**). The sixth row shows a single representative FOD from a region of low fibre density (**c**)

Figure 3 shows an evaluation of the three reconstructed data sets in a coronal slice at the crossing of the CST and corpus callosum. The slice position was selected to maximise the number of crossing fibres at the rostrum of the corpus callosum. There is clearly improved delineation of the fibre bundles seen with increasing angular resolution, consistent with higher precision of fibre tracking in the major tract between the source in the brain stem and the motor strip (top two rows of Fig. 3). Fibres tracking to the contralateral side via the rostrum of the corpus callosum are also demonstrated more clearly, and can be seen to radiate to the contralateral motor strip at higher density.

The bottom four rows in Fig. 3 show an evaluation of data quality using the individual data elements that constitute the processed data set. Each of these are termed FOD (fibre orientation distribution) functions, and are a convenient and accurate way to represent the diffusion information at a single point^12^. The contrast between the FODs in the bundles and the adjacent brain parenchyma is clearly improved with increasing resolution (third row). Specific ROI locations were chosen to represent three different anatomical environments: (a) maximal crossing fibres (fourth row), (b) maximal anisotropy (or aligned fibre orientation) (fifth row) and (c) both low anisotropy and fibre density (i.e., low 'fibre-to-noise' ratio; bottom row).

Profound changes are evident in the appearance of the FODs at each location across the three data sets. For the maximal crossing FOD (a), the relative amplitudes of the third and fourth largest lobes reduced by 5-fold and 15-fold, respectively (64 directions vs 1150 directions), indicating that these higher-order lobes were largely spurious. For the maximally anisotropic FOD in the corpus callosum (b), the second and third largest lobes dramatically decreased (from 20% of peak FOD amplitude in 64-direction to <1% in the 1150-direction data). For the low axonal density FOD (c), the integral of all lobes decreased by 7-fold (64 directions vs 140 directions) and by 32-fold (64 directions vs 1150 directions). Supplemental Fig. 1 shows FODs from a 3×3 region at the same locations, and generated similar changes in the group averages of FOD lobe magnitudes.

### General tracking performance

In order to evaluate general tracking performance, we performed unconstrained tracking on the three bundles from single seed volumes. In these cases, the only reason for exclusion of a streamline was a change in angle greater than our threshold of 45° occurring within the minimum allowed track length of 20 mm. The number of required attempts to generate 1 million streamlines varied no more than 6% for the CB or 1% for CST. For UF tracking, the 1150-direction data required approximately 30% fewer attempts than the 140- and 64-direction data sets, likely a consequence of the tightly curved shape of this tract (Supplemental Table 2).

### Evaluating tracking performance and spurious fibre generation

Table 1 shows the numbers of dominant and non-dominant streamlines for each bundle and angular resolution. Tracking dominant to non-dominant ratios in the 1150-direction data set were much higher compared to the 64-direction data set. The magnitude of this improvement appeared to be dependent on the apparent complexity of the tract (in terms of length and curvature); for instance, the CB had the greatest improvement (a 34-fold increase in the ratio of dominant to non-dominant fibres), while the improvements in the UF and CST were less striking (5.7-fold and 2.1-fold increases, respectively). The 140-direction data set also showed substantial improvements compared to the 64-direction data for UF (5.8×), CST (1.8×) and CB (16.2×).

**Table 1:** Dominant and non-dominant streamlines at each level of angular resolution

Tract	Angular resolution	Dominant bundle (%)	Non-dominant bundle (%)	Dominant:non-dominant
UF	1150	837,371 (84)	162,629 (16)	5.1
	140	839,814 (84)	160,186 (16)	5.2
	64	464,930 (46)	535,070 (54)	0.9
CB	1150	988,846 (99)	11,154 (1)	88.9
	140	977,202 (98)	22,798 (2)	42.3
	64	722,065 (72)	277,935 (28)	2.6
CST	1150	785,227 (79)	214,773 (21)	3.7
	140	762,123 (76)	237,877 (24)	3.2
	64	636,679 (64)	363,321 (36)	1.8

**Fig. 4:**
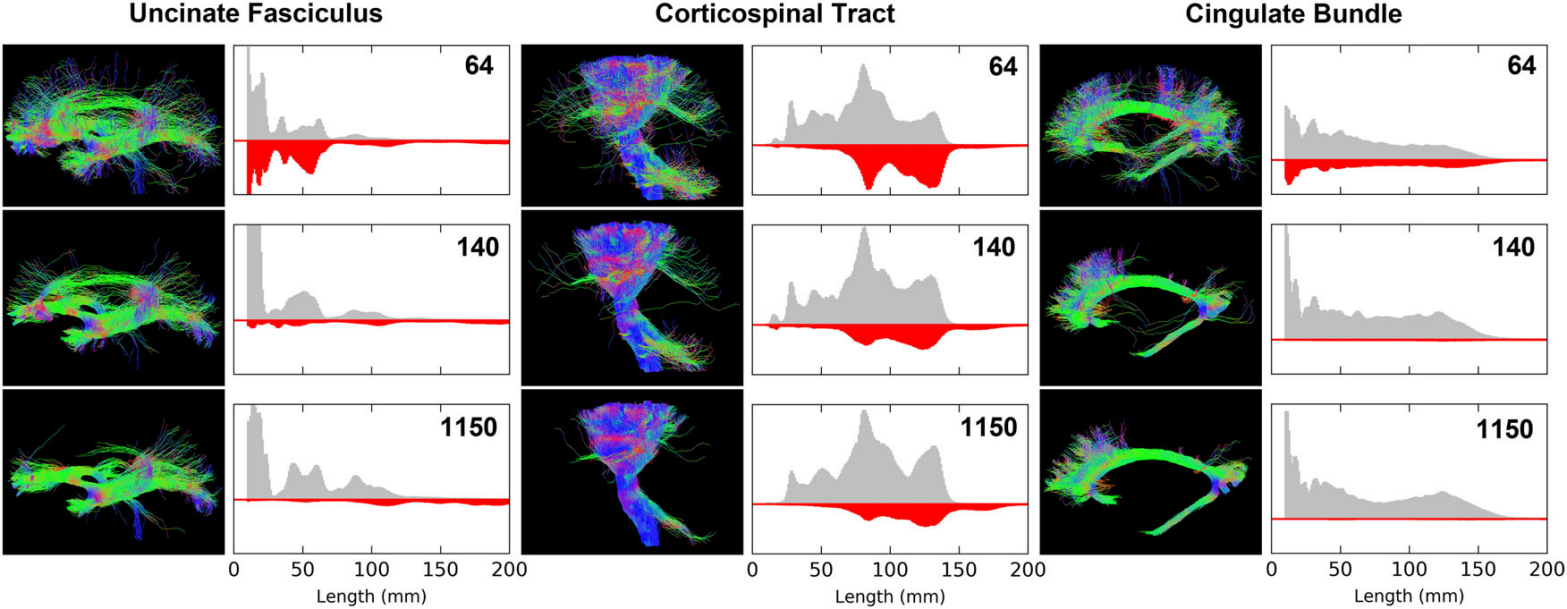
Evaluation of tracking accuracy using unconstrained tracking. Tractographic images are presented with paired histograms of streamline length for each bundle and at each angular resolution. The grey histogram shows the streamlines, which stayed within the master tract (dominant) and the visible red area represents the non-dominant streamlines

Figure 4 shows unconstrained tractography and matching histograms of streamline length for the three bundles. Dramatic reductions in the non-dominant streamlines were seen with increasing resolution, with a general redistribution of streamlines from the non-dominant to the dominant tracks. The peaks in the histograms are increasingly clearly defined with higher angular resolution, a consequence of each tract being more precisely defined by the fibre tracking and so corresponding better to true anatomical features. The impact of improved tracking is particularly evident in the UF, where 30% of all fibres in the non-dominant streamlines are <2.5 cm in length at 64 directions, compared to only 1% of non-dominant fibres in the 1150-direction data. Improved definition of dominant longer fibres is also striking for the 1150-direction data, where 24% of fibres are >7.5 cm compared to only 11% for both 64 directions and 140 directions (Supplemental Table 3).

While these improvements are compelling, they also reveal the complexity of measuring improvements in tracking accuracy, which is both length-specific and tract-specific. For example, in the CB, the non-dominant streamlines are spread uniformly along the range of lengths while, for the CST, there are very few short non-dominant streamlines, but many long ones. For UF and CB, high numbers of shorter fibres are seen in 64-direction data, with a marked decrease at higher angular resolution.

### Evaluating the efficiency of source-to-target tracking

Figure 2 shows the results of an analysis designed to illustrate the impact of source-to-target tracking across the data sets. Focal tracts were defined using the fibre density maps created above, and tracking efficiency was evaluated from the number of streamlines out of 1 million source seeds that reached the target ROIs. The percentage increase in streamline count over the 64-direction data for the UF, CB and CST was 30%, 43% and 69%, respectively, for 64 directions vs 1150 directions, with approximately half of this improvement captured using the 140-direction data (improvement from 64-direction 22–24%; Supplemental Table 4). A corresponding test, counting the number of seed attempts at a source, to generate 1000 successful streamlines to the corresponding target confirmed these ratios of improvement (Supplemental Table 4).

### Track complexity and branching along the cingulate

To evaluate changes in the representation of branching complexity, we looked at fibre numbers along the CB. There were no overt differences between data sets in streamline count at points 1–4 along the CB (Supplemental Table 5). Given that the total streamline count did not vary greatly between data sets, as presented in Fig. 4, this may be expected. However, the number of branching streamlines measured at the frontal pole was considerably greater for the 1150-direction data set with 46 and 72% increases in streamline count over the 140- and 64-direction data sets, respectively. This may reflect a greater capacity of the high-resolution data to detect decussating fibres. This also demonstrates that, in relatively uniform anisotropic bundles such as the CB, the number of diffusion directions is not a large factor for tracking performance.

### Generalisation of results to the CDCP cohort

In order to generalise our findings, we employed data from 53 normal individuals from the CDCP Cohort that utilised the same 140-direction acquisition scheme. The 64-direction data set was extracted from the 140-direction data as described above. We observed increased streamline counts when averaged across for the source-to-target analysis in the 140-direction data set compared to the 64-direction data set. The UF showed on average a greater improvement across the CDCP cohort between 64-direction and 140-direction data (56%, *t* = 2.193, *p* = 0.035) than in the individual brain (24%). The magnitude of the improvement seen in the CB and CST tracts was similar (CB: 22%, *t* = −3.077, *p* = 0.004; CST: 17%, *t* = 1.755, *p* = 0.090) although variation in CST tracking was much higher across the group, decreasing the significance level.

The comparison of tracking accuracy for the CB confirmed the general trends seen for the individual brain. At each length increment, the streamline count decreased; but to an increasingly lesser extent in the 1150-direction data set. The increase in streamline count at intermediate points 1, 2, 3 and 4 between 64 and 140 directions was 6%, 12%, 21% and 50%, respectively.

## Discussion

Recent improvements in MRI gradient technology, acquisition techniques and post-processing have greatly enhanced our ability to meaningfully measure and analyse brain networks; however, there has been no real translation of these advances into improvements in diagnosis for psychiatric conditions. The current data demonstrate that a 'best-case' optimised implementation of these advances confers substantial improvements in fibre tracking. The magnitude of the improvements is considerable, and strongly suggest that studies investigating treatment effects in psychiatry should use advanced high-angular resolution dMRI protocols similar or superior to the 140-direction protocol we tested. It remains to be shown whether dMRI is ready for individualised clinical decision-making, however the magnitude of the improvements we demonstrate, the reduction of biased or spurious tracking and the likely future reduction in acquisition times mean that this is now a very real possibility.

The data we present are the highest single acquisition dMRI to date, with an angular resolution of nearly twice the maximum number of diffusion directions than has previously been reported in the literature. Previously, the highest resolution data set was 515 directions across a single coronal slice (3.6 mm isotropic voxels, max maximum *b* value = 17,000, one channel head coil, time = 25 min^13^), and in the whole brain (2 mm isotropic voxels, maximum *b* value = 6000 mm/s^2^, 32-channel head coil, parallel factor = 2; time = 100 min^14,15,]^). Over 18 scanning sessions, Froeling et al.^16^ acquired close to 2000 diffusion directions in a single subject with *b* values of up to 9000 s/mm^2^, sampled in configurations of five shells and two Cartesian grids at an in-plane resolution of 2.5 mm^2^ (22.5-h acquisition time).

We were able to show an improvement of up to 34-fold greater proportion of 'dominant' streamlines across three important white matter bundles using a gold standard 1150-direction protocol, compared to a conventional 64-direction protocol. Importantly, much of this improvement was also captured by a clinically practical 9-min 140-direction acquisition (a maximal 17-fold increase compared to 64-direction data in the CB). We also showed a large improvement in tracking efficiency, which generalised across a cohort of 53 individuals. Our data show that the improvement in tracking depends very much on the anatomy, which implies that there is the potential for tract-specific bias in conclusions derived from lower angular resolution data.

The advances combined in our current approach have high practical relevance because they enable a higher sensitivity to detect aberrant networks or specific white matter tract abnormalities, potentially pushing the sensitivity toward levels enabling a personalised medicine approach. One condition that could potentially benefit is depression, globally the second largest source of years lost to disability. There are no clinically useful prognostic tools to guide treatment choices in depression^17^; however, it is now accepted that specific neural network abnormalities play a central role in the development and maintenance this disorder^7,18,19^. Many candidate biomarkers have already been identified using dMRI, and are plausible candidates for a clinically actionable biomarker^4,20,21^. Clearly, improved sensitivity in dMRI would improve our ability to detect these aberrant networks outside of highly powered large-cohort trials.

This study does have some limitations. First, a more generalised approach involving the whole brain is needed. This would provide better information regarding region-to-region variations in track strength and in spurious track generation. While angular resolution is the limiting factor at the standard current spatial resolution of 1.5–2.5 mm, it is possible that this relatively coarse spatial resolution may become more limiting at the resolution we acquired. Future work is needed with a greater number of subjects, and the inclusion of clinical subjects will permit investigation of the impact on the psychiatric diagnosis, treatment and outcome. Our conclusions are likely quite conservative, since our low-resolution data set was extremely high quality, benefitting from optimal acquisition and processing.

## Conclusion

We acquired the highest-ever angular resolution dMRI in the human brain in an optimised single 90-min acquisition. We demonstrated a maximum accuracy improvement over a conventional acquisition of up to 34-fold, and maximum 17-fold improvement using a more practical shorter 9-min variant. Further work is needed to demonstrate how this improved sensitivity translates to the detection of pathology.

## Electronic supplementary material

Supplemental Tables 1-5

Supplemental Figure 1
