# Supplementary material for: Toward personalised diffusion MRI in psychiatry: improved delineation of fibre bundles with the highest-ever angular resolution in vivo tractography
Source: Transl Psychiatry. 2018 Apr 25;8:91. doi: 10.1038/s41398-018-0140-8 (PMC5915595; doi:10.1038/s41398-018-0140-8)
Supplement: Supplementary file 1 — Supplemental Tables 1-5 [file 41398_2018_140_MOESM1_ESM.docx]

*Supplemental Table 1. Parameters for each diffusion-weighted magnetic resonance imaging sequence.*

| Sequence | # directions | # shells | Directions in each shell | B-value per shell | # non-diffusion volumes |
| --- | --- | --- | --- | --- | --- |
| 1 | 140 | 3 | 25, 40, 75 | 700, 1000, 2800 | 8 |
| 2* | 6 | 1 | 6 | 2800 | 1 |
| 3 | 123 | 3 | 41, 41, 41 | 500, 850, 1200 | 5 |
| 4 | 124 | 2 | 62, 62 | 1400, 1600 | 5 |
| 5 | 123 | 3 | 41, 41, 41 | 1800, 2000, 2500 | 7 |
| 6 | 127 | 8 | 15, 16, 16, 16, 16, 16, 16, 16 | 500, 850, 1200, 1400, 1600, 1800, 2000, 2500 | 7 |
| 7 | 127 | 8 | 16, 15, 16, 16, 16, 16, 16, 16 | 500, 850, 1200, 1400, 1600, 1800, 2000, 2500 | 8 |
| 8 | 128 | 8 | 16, 16, 16, 16, 16, 16, 16, 16 | 500, 850, 1200, 1400, 1600, 1800, 2000, 2500 | 8 |
| 9 | 129 | 8 | 16, 17, 16, 16, 16, 16, 16, 16 | 500, 850, 1200, 1400, 1600, 1800, 2000, 2500 | 8 |
| 10 | 129 | 8 | 17, 16, 16, 16, 16, 16, 16, 16 | 500, 850, 1200, 1400, 1600, 1800, 2000, 2500 | 9 |
| TOTAL** | **1150** | **11***** |  |  | **65** |
|  |  |  |  |  |  |
| Datasets compared | | | | | |
|  |  |  |  |  |  |
| 1 | 64 | 1 | 64 | 2800 | 4 |
| 1 | 140 | 3 | 25, 40, 75 | 700, 1000, 2800 | 8 |
| 1 to 10** | 1150 | 11 | 121, 25, 121, 40, 121, 142, 142, 121, 121, 121, 75 | 500, 700, 850, 1000, 1200, 1400, 1600, 1800, 2000, 2500, 2800 | 65 |

*=reverse blipped sequence
**=excluding reverse blipped sequence
***due to shells from different sequences with same b-value but different directions. Hence, 11 shells (b-values=500,700 850,1000,1200,1400,1600,1800,2000,2500,2800).

*Supplemental Table 2. General tracking performance: the number of required tracking attempts to successfully generate 1,000,000 unconstrained streamlines for each tract.*

| Angular Resolution | UNCinate fasciculus | cingulate  bundle | cerebrospinal tract |
| --- | --- | --- | --- |
| 1150 | 1,693,116 | 1,605,205 | 1,000,568 |
| 140 | 2,350,351 | 1,703,740 | 1,010,087 |
| 64 | 2,451,557 | 1,654,650 | 1,011,210 |

*Supplemental Table 3. Comparison of streamline lengths in the uncinated fasciculus. Cutoffs at 2.5 cm and 7.5 cm were defined at the troughs seen in Figure 3.*

| Angular Resolution | proportion above 2.5 cm (%) | proportion below 2.5 cm (%) | proportion above 7.5 cm (%) | proportion below 7.5 cm (%) |
| --- | --- | --- | --- | --- |
| 1150 | 68.1 | 31.8 | 35.2 | 64.7 |
| 140 | 46.1 | 53.8 | 19.0 | 80.9 |
| 64 | 60.6 | 39.3 | 15.4 | 84.5 |

*Supplemental Table 4. Source-to-target tracking: the number of required tracking attempts to successfully generate 1,000 streamlines for each tract.*

| Number of Required tracking attempts | | | |
| --- | --- | --- | --- |
| Angular Resolution | **UNCINATE FASCICULUS** | **CINGULATE  BUNDLE** | **CEREBROSPINAL TRACT** |
| 1150 | 132,430 | 181,035 | 1,531,972 |
| 140 | 159,874 | 168,088 | 5,879,117 |
| 64 | 197,941 | 234,426 | 5,411,441 |
| number of streamlines reaching the target | | | |
| Angular Resolution | **UNCINATE FASCICULUS** | **CINGULATE  BUNDLE** | **CEREBROSPINAL TRACT** |
| 1150 | 5,993 | 6,512 | 269 |
| 140 | 4,582 | 5,073 | 102 |
| 64 | 4,185 | 3,705 | 84 |

*Supplemental Table 5. Tracking at intermediate points along the cingulate bundle: the number of streamlines reaching the target from the posterior cingulate. Points 1-4 are progressively further from the posterior cingulate, uniformly spaced up to the sub-genual anterior cingulate. Point 3A is at the frontal pole and thus represents fibres branching off the cingulate.*

| Angular Resolution | 1 | 2 | 3 | 4 | 3a (Branching) |
| --- | --- | --- | --- | --- | --- |
| 1150 | 256,598 | 151,054 | 51,715 | 6,442 | 2,095 |
| 140 | 287,324 | 153,386 | 46,251 | 5,600 | 1,122 |
| 64 | 286,843 | 150,138 | 52,115 | 4,980 | 594 |
