# Supplementary figures and images for: Toward personalised diffusion MRI in psychiatry: improved delineation of fibre bundles with the highest-ever angular resolution in vivo tractography
Source: Transl Psychiatry. 2018 Apr 25;8:91. doi: 10.1038/s41398-018-0140-8 (PMC5915595; doi:10.1038/s41398-018-0140-8)

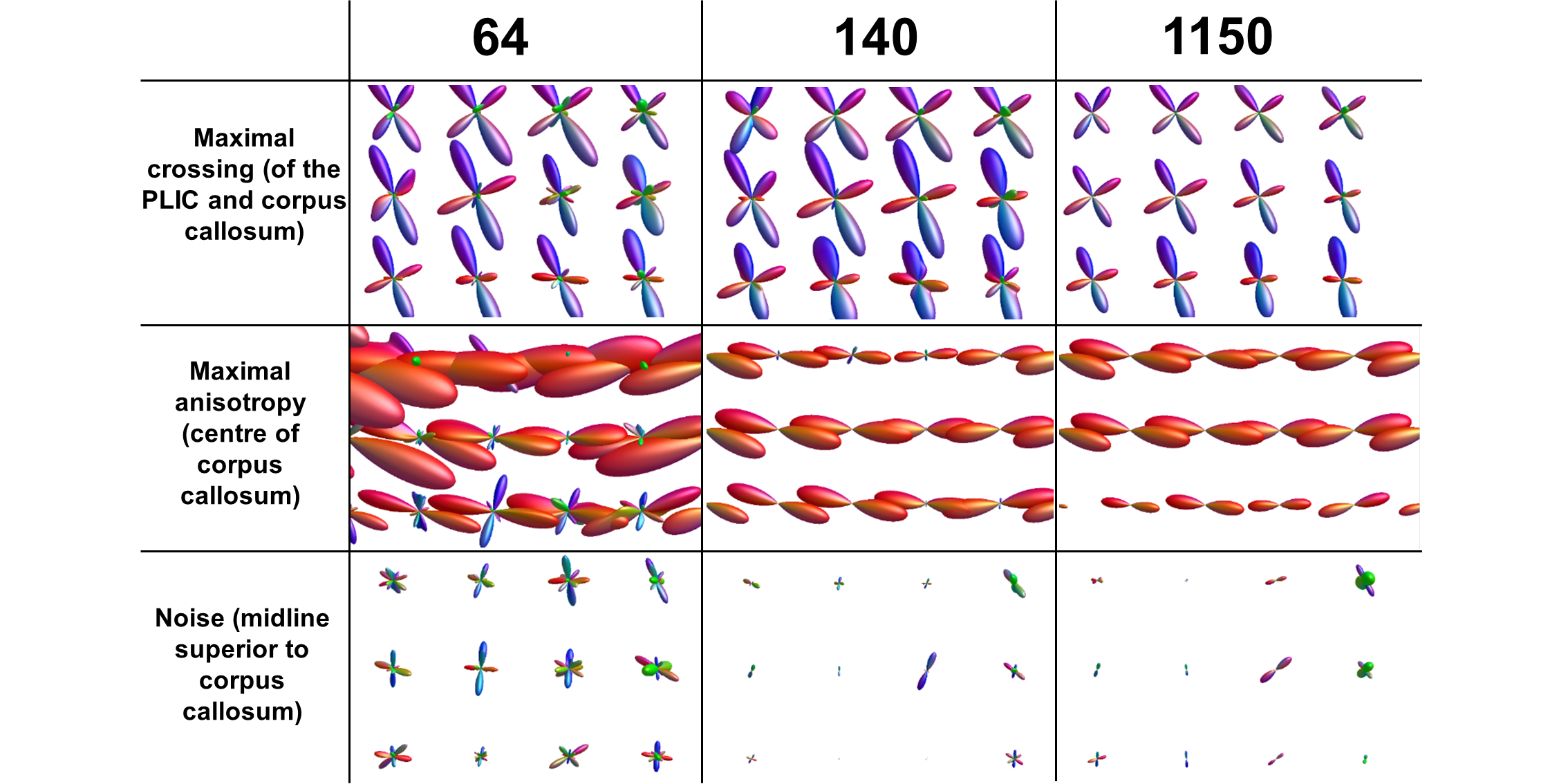

Supplement: Supplementary file 2 — Supplemental Figure 1 [file 41398_2018_140_MOESM2_ESM.tif]
